# High-resolution whole-heart contrast-enhanced coronary MRA in 5 minutes with self-navigation and 100% gating efficiency

**DOI:** 10.1186/1532-429X-16-S1-O80

**Published:** 2014-01-16

**Authors:** Jianing Pang, Qi Yang, Kuncheng Li, Yi He, Zhanming Fan, Bin Sun, Fabio S Raman, Mark A Ahlman, David Bluemke, Xiaoming Bi, Jing An, Daniel S Berman, Debiao Li

**Affiliations:** 1 Biomedical Imaging Research Institute, Cedars-Sinai Medical Center, Los Angeles, California, USA; 2 Radiology and Biomedical Engineering, Northwestern University, Chciago, Illinois, USA; 3 Department of Radiology, Xuanwu Hospital of Capital Medical University, Beijing, China; 4 Department of Radiology, Anzhen Hospital, Capital Medical University, Beijing, China; 5 Fujian Medical University Union Hospital, Fuzhou City, China; 6 Radiology and Imaging Sciences, National Institutes of Health, Bethesda, Maryland, USA; 7 Molecular Biomedical Imaging Laboratory, National Institutes of Health, Bethesda, Maryland, USA; 8 Siemens Medical Solutions, Los Angeles, California, USA; 9 Siemens Medical Solutions, Beijing, China

## Background

Contrast-enhanced (CE) whole-heart coronary MRA is a promising technique for CAD detection [1]. However, the current imaging time is relatively long and variable, and the spatial resolution is limited. The aim of this work is to develop and evaluate a 3D projection reconstruction (3DPR) based CE coronary MRA technique that achieves (1.0 mm)^3 ^spatial resolution and 5-minute scan time, which has been previously validated on non-contrast coronary MRA [2]. Similar to the previous work, we compare the apparent SNR of two undersampling levels with 10,000 and 20,000 radial projections to explore the impact of undersampling on image quality in the context of CE imaging.

## Methods

We employed a 3-bolus contrast injection scheme: the first two boluses are in the size of 0.05 mmol/kg at 4 ml/s intended for the stress and rest perfusion scans, and the remaining 0.10 mmol/kg is injected right before the coronary MRA at the same rate. We used an ECG-gated, fat-saturated, inversion-recovery prepared spoiled gradient-echo sequence with 3DPR k-space trajectory for free-breathing data acquisition with self-navigated motion correction and integrated non-Cartesian sensitivity encoding acceleration [2]. We performed healthy volunteer studies (N = 10) to compare the image quality at two undersampling levels, 10,000 projections and 20,000 projections, which correspond to scans times of around 5 and 10 minutes, respectively. The comparisons are made on apparent signal-to-noise ratio (aSNR), measured as the ratio between the average signal within a blood ROI and the standard deviation within a background ROI.

## Results

**Figure 1:**
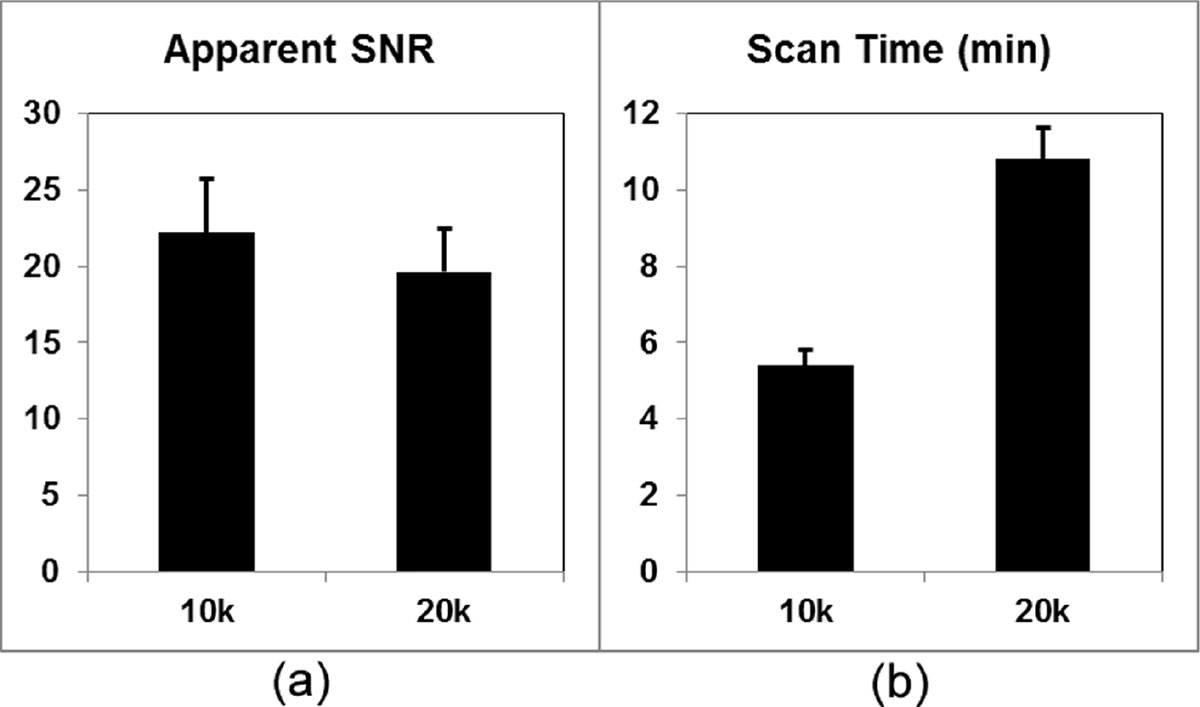
**(a) the 10,000 projection images, acquired during the first 5 minutes of contrast injection, show significantly higher aSNR than the 20,000 projection images (P < 0.05); (b) it takes an average time of 5.4 minutes to acquire the 10,000 projection images, half of the time to acquire 20,000 projections**.

**Figure 2:**
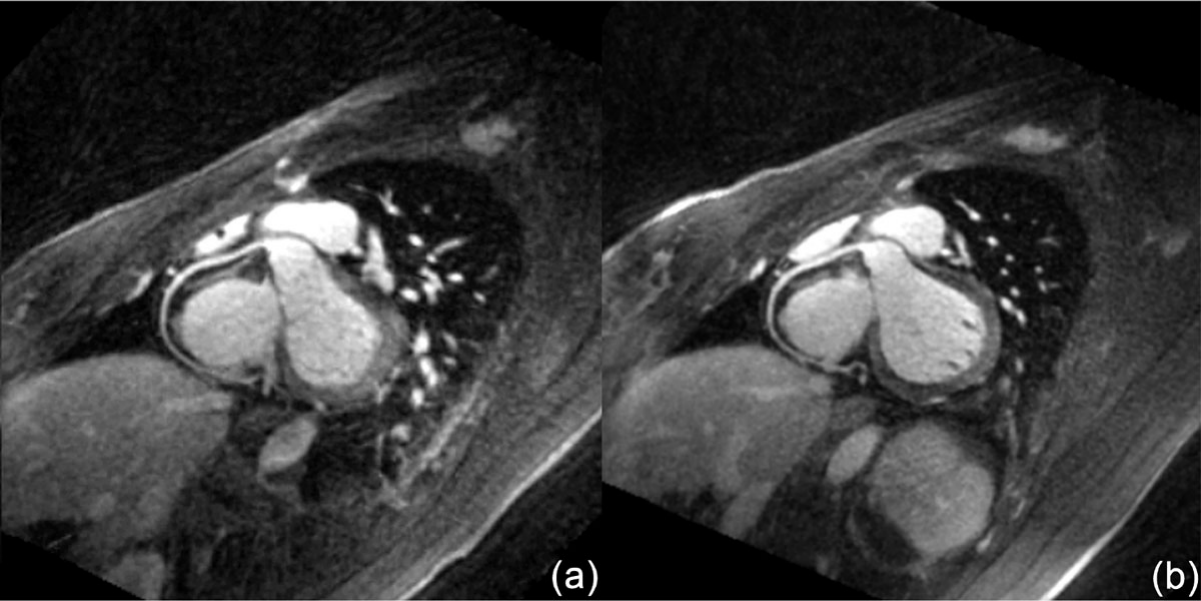
**Example RCA visualizations**. (a) 10,000 projections; (b) 20,000 projections

As shown in Figure 1, the average scan times of the 10,000 and 20,000 projection images were 5.4 ± 0.4 and 10.8 ± 0.8 minutes, and the average aSNR values were 22.20 ± 3.51 and 19.61 ± 2.81, respectively. The 10,000 projection images show significantly higher aSNR values due to the higher contrast concentration on the earlier stage of the acquisition. Additionally, the employed non-Cartesian acceleration suppressed the streaking artifacts, therefore maintained the aSNR of the undersampled image despite a 50% scan time reduction. Example images are shown in Figure 2.

## Conclusions

We have developed a CE coronary MRA technique that delivers good image quality at (1.0 mm)^3 ^spatial resolution with scan time of 5 minutes. Further investigations are warranted on subjective image quality evaluation, protocol optimization, and CAD patient studies.

## Funding

NIH Grant Numbers: HL38698, EB002623.

## References

[B1] Yang Circ Cardiovasc Imaging 2012

[B2] Pang ISMRM 2013 1295

